# Yes, I can - maybe … Effects of placebo-related instructions on neuroregulation in children with ADHD

**DOI:** 10.1007/s00702-020-02193-z

**Published:** 2020-05-10

**Authors:** Holger Gevensleben, David Schmiedeke, Hartmut Heinrich, Aribert Rothenberger

**Affiliations:** 1 Clinic for Child and Adolescent Psychiatry and Psychotherapy, University Medical Center Göttingen, von-Siebold-Str. 5, D37075 Goettingen, Germany; 2 NeuroCare Group, Munich, Germany; 3 Research Institute Brainclinics, Nijmegen, The Netherlands; 4 kbo-Heckscher-Klinikum, Munich, Germany; 5 Department of Child and Adolescent Mental Health, University Hospital Erlangen, Friedrich-Alexander-University Erlangen-Nuremberg, Erlangen, Germany

**Keywords:** Attention-deficit/hyperactivity disorder (ADHD), Placebo, Neurofeedback, Theta-beta-training, Neuroregulation

## Abstract

Neurofeedback training (NFT) provides strategies for children with ADHD to achieve changes on the neurophysiological and behavioral level to attenuate ADHD symptoms. We assume self-efficacy (an “active attitude”), a core variable in successful NFT. In a randomized, double-blind controlled study, we investigated the impact of a “placebo instruction” on the EEG-regulation performance during two sessions of Theta-Beta-NFT in children with ADHD (age 8–12 years). Children receiving the information “this might be a placebo-training” showed inferior neuroregulation (*n* = 10) compared to children receiving a standard instruction (*n* = 12). Results of our pilot study are discussed in view of factors necessary for a valid training (ensuring maximal self-efficacy in the participants) and the fidelity of placebo-controlled trials in NFT-research.

**Electronic supplementary material:**

The online version of this article (10.1007/s00702-020-02193-z) contains supplementary material, which is available to authorized users.

## Introduction

EEG-neurofeedback training (EEG-NFT) allows inducing changes in neuronal regulation and behavior in children with ADHD. In several randomized-controlled trials (either comparing NFT to a control group or between different NF protocols), it could be demonstrated that NFT is more than just a “placebo phenomenon" (Heinrich et al. 2019). However, there still exists a debate how these neurophysiological changes may translate into clinically relevant symptom reductions (Gevensleben et al. 2014a).

In general, there are core “top down” variables unfolding an impact on the outcome of psychotherapeutic approaches (e.g., treatment fidelity and self-efficacy; Wampold & Imel, 2015), likely impaired by placebo conditions (Gevensleben et al. 2014a, b). Placebo expectations might even induce neurophysiological changes interfering with NFT neuroregulation (Kober et al. 2018). Hence, an “active attitude” towards the objectives of NFT should, therefore, improve neuronal regulation during NFT and, thus, enhance the translation into the expected behavioral changes. However, trials inducing a placebo-expectation in children with ADHD often failed in showing specific effects of NFT (e.g., Van Dongen-Boomsma et al. 2013). The notion of placebo control in NFT (and in psychotherapy overall) demands the further research. Following this notion, the objective of this short-term NFT pilot study in children with ADHD was to test experimentally if manipulating the certitude of control about the upcoming neurofeedback (real vs. potentially placebo) may influence neuroregulation in Theta–Beta NFT. We expected a gain in regulation capability even in only two sessions (enhancement of beta and reduction of theta) with diminished regulation capability in the “placebo-control” group (due to a lower certitude of control following the instruction that the feedback might be a placebo condition).

## Methods

In this short-term study (within a series of investigations testing methodical NF aspects), 22 children with ADHD (according to DSM-IV diagnostic criteria) whose families had contacted our outpatient department for NFT were included. Participants received two sessions of Theta–Beta NFT (ahead of a regular 30 sessions NFT). Children taking methylphenidate medication were drug-free for at least 24 h before the sessions. Both parameters (theta, beta) were presented separately on different spatial locations on the screen of the NF system (by two separate bars). Randomization: participants were assigned to the training slots in order of their registration to our neurofeedback-waiting-list (even numbers = “standard instruction” vs. uneven numbers = “placebo instruction”). The experimental group received a standard instruction to the standard NFT (self-control instruction; SCI; see Gevensleben et al. 2009) while the control group received a "potential-placebo instruction” to the standard NFT including, that participants could either control the bars on the screen with their brain activity (called “real training”) or that they could not, but instead the bars would move randomly (called “sham training”). The instruction was applied in written form and additionally verbally explained. None of the children gave any hints not to understand this notion. (“I can control these bars with my brain” vs. “I cannot control these bars with my brain”).

The NF-trainers were blind to the instruction (i.e. did not receive information which instructions a child had received by the trial coordinator) and motivated all participants equally to actively engage in the training. Both groups were instructed to get into an alert and focused, but relaxed state, and to find individual strategies to control the feedback animation (bars). Both NFT sessions contained two trials of contingent feedback of neuronal activity (block 1: trial 1 + 3) and two trials of delayed feedback (block 2: trial 2 + 4), each trial lasted for 300 s.

For NFT, the program “Self-Regulation and Attention Management” (SAM, Gevensleben et al. 2009) was used. Children had to find out what was depicted on a picture by uncovering puzzle pieces. The better the neuregulation (i.e., reducing Theta and increasing Beta activity) was, the faster the puzzle pieces were uncovered. NFT was provided based on the signal at Cz (referenced to FCz), for details regarding recording, artifact processing, feedback calculation, and presentation, see Van Doren et al. 2017. Theta and Beta values were referred to a 3 min baseline recording. For each trial, mean Theta and Beta changes from baseline over a trial (effective values) were subjected to ANOVAs with between-subject factor “Group” (SCI vs. PPI) and within-subject factors “Session” (first vs. second), “Condition” (contingent vs. delayed feedback) and “Time” (first vs. second trial), i.e., variables related to the NFT that could have an impact on neuroregulation.

## Results

Experimental group (“self-control instruction”, SCI; *n* = 12) and control group (”potential-placebo instruction “, PPI; *n* = 10) did not differ significantly regarding age, sex, in parent ratings on ADHD symptoms and associated domains and regarding baseline theta and beta EEG (see Table 1; for more details: Table S-1).

**Table 1:** Demographic, behavioral, and EEG baseline characteristics of the self-control instruction group and the potential-placebo instruction group

	Self-control instruction (*n* = 12)	Potential-placebo instruction (*n* = 10)	Statistics
Age (months)	117.0 (15.6)	111.6 (19.2)	*t*(20) = 1.06
Sex (boys/girls)	10/2	8/2	^*χ*2^ = 0.04
ADHD rating scale (FBB-ADHS; Döpfner et al. 2008)			
Total score	34.2 (13.2)	31.5 (7.5)	*t*(20) = 0.67
Inattention	17.3 (5.3)	19.3 (4.2)	*t*(20) = 1.05
Hyperactivity/impulsivity	17.3 (8.6)	13.1 (5.9)	*t*(20) = 1.43
Conduct Disorder Rating Scale (FBB-SSV; Döpfner et al. 2008)			
Total score	18.6 (8.3)	16.8 (9.1)	*t*(20) = 0.53
Strengths and Difficulties Questionnaire (SDQ; Woerner et al. 2004)			
Total score	16.8 (4.9)	18.4 (5.8)	*t*(20) = 0.78
EEG–baseline (µV)			
Theta (session 1)	3.30 (0.95)	2.96 (0.66)	*t*(20) = 0.93
Theta (session 2)	3.34 (0.91)	2.98 (0.50)	*t*(20) = 1.11
Beta (session 1)	1.36 (0.70)	1.21 (0.24)	*t*(20) = 0.64
Beta (session 2)	1.33 (0.62)	1.20 (0.20)	*t*(20) = 0.64

Mean values and standard deviations are listed for each group

For all statistical tests, *p* >0 .17

**Fig. 1:**
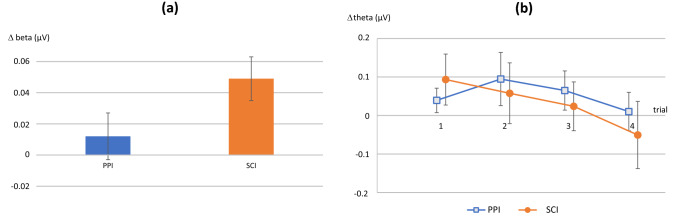
**a** Beta activity (referred to baseline, averaged over both sessions and all trials) is higher in the 'self-control instructions' group (SCI, orange) compared to the 'potentially placebo instructions' group (PPI, blue). **b** Theta activity in the second session (referred to baseline) shows a steeper decline in the SCI group (orange) compared to the PPI group (blue) over the course of the session. Means ± SE are depicted

The ANOVAs with between-subject factor “Group” (SCI vs. PPI) and within-subject factors “Session” (first vs. second), “Condition” (contingent vs. delayed feedback), and “Time” (first vs. second trial) revealed a trend for Group which indicated a better regulation in the SCI group (*F*(1,20) = 3.34; *p* = 0.08: see also Fig. 1). Actually, successful regulation of Beta activity was found only in the SCI group (*F*(1,20) = 16.98; *p* = 0.002; part. *η*^2^ = 0.61) but not in the PPI group (*F*(1,20) = 0.49; *p* = 0.50; part. *η*^2^ = 0.05). A significant interaction Group x Session x Condition (*F*(1,20) = 4.94, *p* = 0.04, part. *η*^2^ = 0.20) was obtained indicating superior regulation capability of the SCI group of trials with delayed feedback across sessions (more pronounced in session 1 vs. session 2).

Regarding regulation of Theta activity, we found a trend for Condition indicating a better regulation for delayed feedback (*F*(1,20) = 3.56; *p* = 0.074; part. *η*^2^ = 0.15, and a significant Condition × Time interaction towards better regulation with delayed feedback in the second trial of each session (*F*(1,20) = 12.46; *p* = 0.002; part. *η*^2^ = 0.38) indicating learning (superior regulation) in the progress of the sessions. Finally, an interaction Group × Session × Condition (*F*(1,20) = 4.69, *p* = 0.04, part. *η*^2^ = 0.19) indicated a better learning with delayed feedback in the SCI group (Fig. 1 and Table S-2).

## Conclusion and discussion

Results indicate that it is possible for children with ADHD to achieve control over a distinct EEG pattern (Theta and Beta activity) already within two sessions and that regulation is superior if feedback is presented in a delayed way (as children may be less distracted by the integration of the feedback information; Johnson et al. 2012).

However, if children were instructed that the feedback could be a placebo condition, regulation capability and/or learning of both EEG parameters (Theta and Beta) was reduced even in our relatively small sample. Hence, questioning the certitude of control by pre-treatment instruction may influence neuroregulation (and probably translation into behavior) in a negative way. Although these results may be different after 25–40 sessions of NFT, they argue for the importance of “active attitude” instructions before (and during) the training and might point to the responsibility of the treatment and the trainer, to assure and improve self-efficacy in the patient if NFT is interpreted as a “skill acquisition”-treatment (Gevensleben et al. 2014a).

It may also have implications for the design of NFT trials as it challenges the use of placebo-control conditions (see also Pigott et al. 2018). Good alternative approaches are at hand (see, e.g., Gevensleben et al. 2014a). Thus, there is no need for treatment instructions which diminish self-efficacy and treatment fidelity even before the first treatment session started.

Limitations: This study included children diagnosed with ADHD who were forwarded to the neurofeedback program of our clinic by local medical specialists and, therefore, represent an ecologically valid sample of outpatient NF children. However, we did not verify diagnosis, assess for comorbidity, actual treatment (e.g., psychotherapy) or intelligence. Therefore, we cannot evaluate whether experimental and control groups differed systematically concerning these variables (despite randomization).

Our preliminary pilot study can be considered a first step to directly test the impact of invalid instructions on performance in NF. Results will have to be confirmed in larger trials. Larger sample sizes and a complete number of treatment sessions are required to further test the validity of a “skill acquisition” model for NFT (Gevensleben et al. 2014b) and evaluate the value of placebo treatment conditions in NF research. However, our first results confirm the assumption that the kind of instructions or settings of an NFT (e.g., placebo control) influence the fidelity of the treatment.

## Electronic supplementary material

Below is the link to the electronic supplementary material.

Supplementary file1 (DOCX 16 kb)

## Data Availability

The data sets used and/or analyzed during the current study are available from the corresponding author on reasonable request.
