# Supplementary material for: Yes, I can - maybe … Effects of placebo-related instructions on neuroregulation in children with ADHD
Source: J Neural Transm (Vienna). 2020 May 10;127(7):1093–6. doi: 10.1007/s00702-020-02193-z (PMC7293682; doi:10.1007/s00702-020-02193-z)
Supplement: Supplementary file 1 — Supplementary file1 (DOCX 16 kb) [file 702_2020_2193_MOESM1_ESM.docx]

**Table S1:** Behavioral/clinical characteristics (parent ratings) and achievement motivation (self-ratings) of the self-control instruction group and the potential-placebo instruction group. The table provides additional information to Table 1 (in the manuscript).

|  | **Self-control  instruction (n=12)** | **Potential-placebo instruction (n=10)** | **t-tests** |  |
| --- | --- | --- | --- | --- |
| ADHD-behavior rating scale (FBB-ADHS; Döpfner et al. 2008) | | | |  |
| Total score | 34.20 (13.21) | 31.50 (7.50) | t(20) = .668 |  |
| Inattention | 17.27 (5.34) | 19.25 (4.20) | t(20) = .051 |  |
| Hyperactivity/ impulsivity | 17.27 (8.64) | 13.08 (5.93) | t(20) = 1.427 |  |
| Conduct disorder behavior rating scale (FBB-SSV; Döpfner et al. 2008) | | | |  |
| Total score | 18.60 (8.34) | 16.83 (9.14) | t(20) = .524 |  |
| Strengths and Difficulties Questionnaire (SDQ; Woerner et al. 2008) | | | |  |
| Total score | 16.80 (4.93) | 18.42 (5.82) | t(20) = .781 |  |
| Emotional problems | 3.20 (2.04) | 3.33 (2.90) | t(20) = .140 |  |
| Conduct problems | 6.40 (2,75) | 7.08 (2.54) | t(20) = .664 |  |
| Hyperactivity | 3.80 (2.68) | 3.75 (2.22) | t(20) = .564 |  |
| Peer problems | 3.73 (2.02) | 4.33 (2.02) | t(20) = .734 |  |
| Prosocial behavior | 6.53 (1.85) | 5.92 (2.50) | t(20) = .360 |  |
| Achievement motivation (SELLMO; Spinath et al., 2012), self-ratings | | | |  |
| Achievement goals: School | 29.86 (6.41) | 32.09 (8.35) | t(20) = .758 |  |
| Achievement goals: Approaching | 22.36 (4.80) | 22.00 (6.77) | t(20) = .154 |  |
| Achievement goals: Avoidance | 21.64 (6.38) | 20.73 (8.67) | t(20) = .304 |  |
| Avoiding work | 24.50 (7.20) | 22.82 (9.56) | t(20) = .502 |  |

**Table S2:** Theta and beta baseline values and change scores in neurofeedback trials (deviation from baseline). Trials 1, 3: contingent feedback; trials 2,4: delayed feedback). Feedback electrode: Cz (vs. Fcz), unit: µV

|  | **Theta** | **Beta** |  |
| --- | --- | --- | --- |
|  | Mean (SD) | Mean (SD) |  |
| **Group: Self-control instruction (SCI; n=12)** | | |  |
| Session 1 | | |  |
| Baseline | 3.296 (0.950) | 1.358 (0.699) |  |
| Trial 1 | 0.1647 (0.1648) | 0.0381 (0.0950) |  |
| Trial 2 | 0.1858 (0.2037) | 0.0438 (0.0392) |  |
| Trial 3 | 0.2436 (0.2674) | 0.0646 (0.0769) |  |
| Trial 4 | 0.1146 (0.2444) | 0.8670 (0.1051) |  |
| Session 2 | | |  |
| Baseline | 3.338 (0.910) | 1.329 (0.623) |  |
| Trial 1 | 0.0934 (0.2293) | 0.0403 (0.0568) |  |
| Trial 2 | 0.0577 (0.2738) | 0.0569 (0.0600) |  |
| Trial 3 | 0.0238 (0.2297) | 0.0284 (0.0393) |  |
| Trial 4 | -0.0507 (0.3026) | 0.0367 (0.0593) |  |
| **Group: Potential-placebo instruction (PPI; n=10)** | | |  |
| Session 1 | | |  |
| Baseline | 2.963 (0.660) | 1.210 (0.244) |  |
| Trial 1 | 0.0651 (0.2808) | 0.0246 (0.1386) |  |
| Trial 2 | 0.1804 (0.4155) | 0.0890 (0.1081) |  |
| Trial 3 | 0.1063 (0.2925) | 0.0075 (0.1118) |  |
| Trial 4 | 0.0383 (0.3084) | 0.0082 (0.1144) |  |
| Session 2 | | |  |
| Baseline | 2.98 (0.500) | 1.196 (0.202) |  |
| Trial 1 | 0.0394 (0.1005) | 0.0146 (0.0775) |  |
| Trial 2 | 0.0947 (0.2184) | 0.0104 (0.0662) |  |
| Trial 3 | 0.0649 (0.1608) | 0.0196 (0.0811) |  |
| Trial 4 | 0.0103 (0.1579) | 0.0026 (0.0585) |  |

**References**

Spinath B, Stiensmeier-Pelster J, Schöne C, Dickhäuser O (2012) Skalen zur Erfassung der Lern und Leistungsmotivation 2., überarbeitete und neu normierte Auflage. Göttingen: Hogrefe.
